# An Improved Approach for Accurate and Efficient Measurement of Common Carotid Artery Intima-Media Thickness in Ultrasound Images

**DOI:** 10.1155/2014/740328

**Published:** 2014-08-18

**Authors:** Qiang Li, Wei Zhang, Xin Guan, Yu Bai, Jing Jia

**Affiliations:** School of Electronic Information Engineering, Tianjin University, 92 Weijin Road, Tianjin 300072, China

## Abstract

The intima-media thickness (IMT) of common carotid artery (CCA) can serve as an important indicator for the assessment of cardiovascular diseases (CVDs). In this paper an improved approach for automatic IMT measurement with low complexity and high accuracy is presented. 100 ultrasound images from 100 patients were tested with the proposed approach. The ground truth (GT) of the IMT was manually measured for six times and averaged, while the automatic segmented (AS) IMT was computed by the algorithm proposed in this paper. The mean difference ± standard deviation between AS and GT IMT is 0.0231 ± 0.0348 mm, and the correlation coefficient between them is 0.9629. The computational time is 0.3223 s per image with MATLAB under Windows XP on an Intel Core 2 Duo CPU E7500 @2.93 GHz. The proposed algorithm has the potential to achieve real-time measurement under Visual Studio.

## 1. Introduction

In 2011, a report by the World Health Organization [1] revealed that cardiovascular diseases (CVDs) are the number one cause of death globally. An estimated 17.3 million people died from CVDs in 2008, representing 30% of all global deaths. The number of people who die from CVDs will increase to 23.3 million by 2030. Therefore, a growing body of studies is looking for an early diagnosis and treatment of cardiovascular diseases, which is crucial to prevent patients from suffering more serious pathologies.

**Figure 1:**
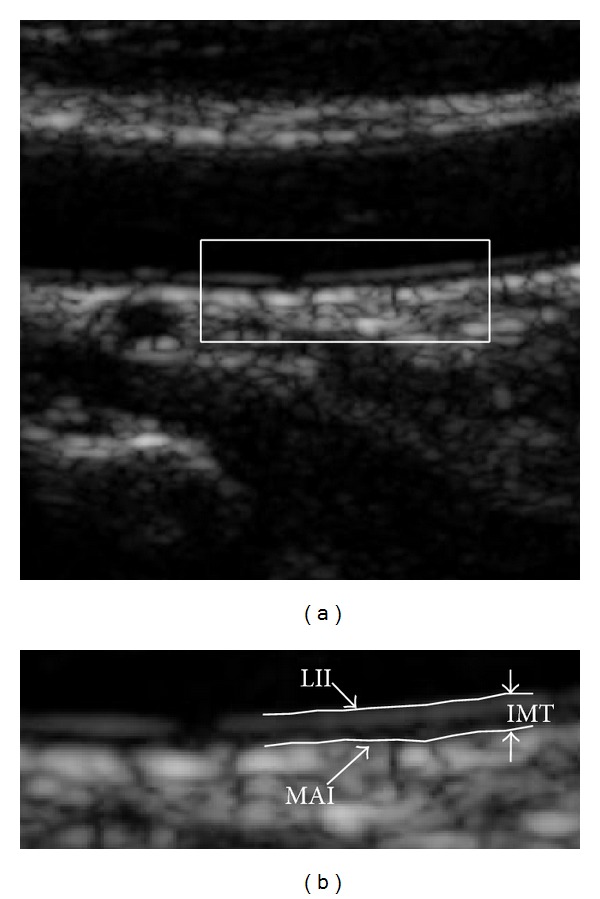
Illustrations of the CCA intima-media complex (IMC) and intima-media thickness (IMT). IMT is the difference between LII and MAI boundaries.

Several large population-based studies [2–5] have shown that the intima-media thickness (IMT) of common carotid artery can serve as an important indicator for cardiovascular diseases at an early stage and can also be used to predict major cardiovascular events. In practice, ultrasound imaging, which has been widely used in medical diagnostic technique due to its noninvasive nature, low cost, and real-time examination, can be applied to measure IMT by visual assessment of the leading edges. The carotid arteries are most suitable for study because of their superficial location, size, and limited movement, and the CCA is easy to image for it is relatively close and parallel to the skin surface. As illustrated in Figure 1, IMT is the distance between the two approximately parallel lines, lumen-intima interface (LII), and media-adventitia interface (MAI), respectively. Conventionally, IMT was measured by manual tracing of the interfaces between tissue layers. This method requires substantial experience, and it is time consuming and varies according to the training, experience, and the subjective judgment of the experts. Therefore, the manual measurements suffer from considerable inter- and intraobserver variability [6, 7].

During the past two decades, efforts have been made by numerous investigators worldwide to try to find an approach for interface detection that is less reliant on human operators; thus many computer-aided methods have been developed to solve this problem. The earliest research on the feasibility study of using B-mode sonographic images to measure the CCA IMT was reported in 1986 [8]. Since then, a wide variety of techniques have been proposed for the IMT measurement, which can be classified into two groups: semiautomatic [9–14] and fully automatic measurement [15–20].

Almost all of the existing methods include two stages: estimating the initial boundaries (LII, MAI) and then adjusting them to their exact locations. For the purpose of the first stage, the semiautomatic techniques need human intervention to select a region of interest (ROI) [11–14] or some points of interest [9, 10]. By contrast, the fully automatic technique can complete the task without any user interaction. Petroudi et al. [18, 19] proposed an algorithm that used variational level set [21] method to segment the ultrasound image into two parts according to image intensity and then estimated the MAI and LII with the boundary of the segmented image. Delsanto et al. [15, 16] developed a completely user-independent algorithm to locate the CCA tunicae as a ROI considering the mean value and variance of image intensity. Molinari et al. [17] described a novel technique called completely automated multiresolution edge snapper (CAMES), which recognized CCA based on a combination of scale-space and statistical classification in a multiresolution framework. The main difference between semi- and fully automatic groups is whether the technique can automatically complete the task of the first stage. In order to achieve the results in the second stage, different methods have been developed for the adjustment, such as active contours [9, 10, 18, 19], Williams-Shah snake [11], dual snakes [14], dynamic programming [12], and dual dynamic programming [13]. A state-of-the-art review on IMT measurement and wall segmentation is presented in [22, 23], and the latest review article of this field is presented in [24].

The aim of this paper is to present an automatic technique for CCA IMT measurement with low computational cost and high accuracy. More specifically, a ROI is extracted at the beginning of the processing, and then the LII and MAI boundaries are estimated within the extracted ROI; finally the estimated boundaries are adjusted to the accurate locations by using improved dynamic programming. The rest of this paper is structured as follows. In Section 2, we will describe the approach in detail.  Section 3 shows the results of our experiments on 100 test images. Finally, Section 4 discusses the accuracy, efficiency, limitations, and advantage of the proposed approach.

## 2. Materials and Methods

### 2.1. Image Dataset

Our dataset consisted of 100 B-mode longitudinal ultrasound images of the CCA from Cyprus Institute of Neurology of Nicosia (Cyprus). These images are acquired by the ATL HDI-3000 ultrasound scanner (Advanced Technology Laboratories, Seattle, USA), which is equipped with 64-element, fine-pitch, high-resolution, 38 mm broadband array, a multielement ultrasound scan head with an operating frequency range of 4–7 MHZ, an acoustic aperture of 10 × 8 mm, and a transmission focal range of 0.8–1.1 cm. Digital images were resized using the bicubic method to standard pixel density of 16.66 pixels/mm. The images were logarithmically compressed and were recorded digitally on a magnetooptical drive at size of 768 × 576 pixels with 256 gray levels. The images were recorded at Cyprus Institute of Neurology and Genetics, in Nicosia, Cyprus, from 42 female and 58 male symptomatic patients aged between 26 and 95 years, with a mean age of 54 years. These subjects were at risk of atherosclerosis and have already developed clinical symptoms, such as a stroke or a transient ischemic attack [11].

In order to remove the textual markers of the images and facilitate the following processing, the original ultrasound images were automatically cropped into a size of 401 × 401 pixels (from 100 to 500 lines and from 200 to 600 columns).

### 2.2. Overview of the Proposed Approach

**Figure 2:**
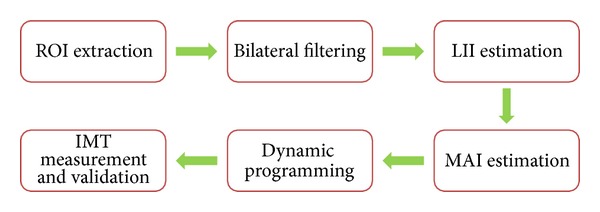
The flowchart of the approach.

The flowchart of the proposed approach is shown in Figure 2. Our approach can be divided into six cascaded steps: (1) the method of template-based matching is utilized for ROI extraction; (2) bilateral filtering is applied to remove noise and artifacts in the ultrasound images; (3) initial LII boundary is estimated within the extracted ROI; (4) initial MAI is estimated based on the edge map and the LII boundary; (5) the estimated boundaries are adjusted to their exact locations with improved dynamic programming; (6) IMT measurement and validation are carried out.

### 2.3. ROI Extraction

The method of template-based matching was applied to extract the ROI. Firstly, a template containing part of the IMC and some lumen and adventitia was constructed according to the pixel density and image contrast. Then, the template was used to find the positions of the IMCs by searching a series of ultrasound images. We call the search image *f*(*x*, *y*), where (*x*, *y*) represents the coordinates of each pixel in the ultrasound image and the template  *T*(*x*_*t*_, *y*_*t*_) with (*x*_*t*_, *y*_*t*_) denotes the coordinates.

In our algorithm, we simply moved the center of the template *T*(*x*_*t*_, *y*_*t*_) over each point (*x*, *y*) on a vertical line in the image and calculated the sum of the absolute differences (SAD) between their pixel intensities. Thus the SAD can be defined as

**Figure 3:**
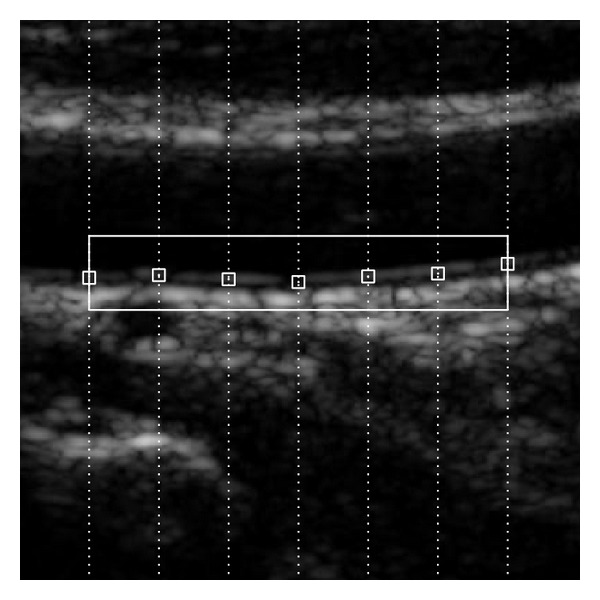
The seven small boxes are the best matches on each vertical line. The rectangular region denotes the extracted ROI.

(1) SAD(x,y)=∑i=x−(Tr−1)/2x+(Tr−1)/2 ∑j=y−(Tc−1)/2y+(Tc−1)/2|f(i,j)−I(i−x+Tr−12,               j−y+Tc−12)|,

where *T*_*r*_ and *T*_*c*_ denote the number of rows and columns of the template, which are assumed as odd integers. In this method the lowest SAD score gives the best estimation for the IMC on that vertical line. Actually, we find seven such points on seven evenly spaced vertical lines.  Figure 3 shows the seven estimated points.

If the image is damaged by speckle noises or artifacts to a large extent, some points may be located at wrong positions. As long as four points or more are positioned correctly, we can achieve a ROI by excluding the wrong points. In practice, the difference in vertical position of the points with each other is computed. If the vertical position of one point has a difference within 2 mm with other three points or more, this point is assumed to be correctly positioned since the CCA IMC almost locates in a horizontal position in the longitudinal CCA ultrasound image.

Among the correctly located points, a rectangular ROI can be extracted with top left and bottom right points, which can be denoted by (min⁡⁡(*x*), *y* − *d*) and (max⁡⁡(*x*), *y* + *d*), respectively, where *d* is a positive constant integer.

### 2.4. Bilateral Filtering

Speckle noises always affect the boundary detection on ultrasound images. Therefore we need to reduce the effect of noise over the extracted ROI at the first place. The bilateral filter [25] has been chosen because it is a simple, noniterative tool for edge preserving as well as noise reducing.

The basic idea underlying bilateral filtering is to combine domain and range filtering, thereby enforcing both geometric and photometric locality. Bilateral filtering can be described as follows:

(2) $$\begin{array}{c} h\left(x\right)=k^{-1}\left(x\right)\overset{\infty }{\underset{-\infty }{\int }}\overset{\infty }{\underset{-\infty }{\int }}f\left(\xi \right)c\left(\xi ,x\right)s\left(f\left(\xi \right),f\left(x\right)\right)d\xi . \end{array}$$

**Figure 4:**
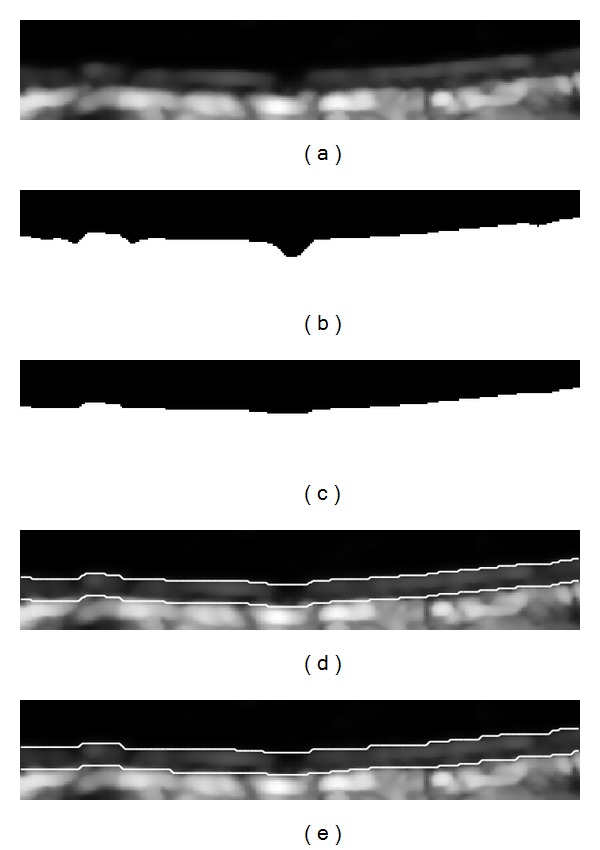
(a) The extracted ROI after bilateral filtering. (b) Binary ROI segmented by threshold. (c) Binary ROI followed by dilation and erosion. (d) The estimated boundaries of LII and MAI. (e) The final boundaries of LII and MAI.

With the normalization

(3) $$\begin{array}{c} k\left(x\right)=\overset{\infty }{\underset{-\infty }{\int }}\overset{\infty }{\underset{-\infty }{\int }}c\left(\xi ,x\right)s\left(f\left(\xi \right),f\left(x\right)\right)d\xi , \end{array}$$

where *c*(*ξ*, **x**) measures the geometric closeness between the neighborhood center **x** and a near point *ξ* and *s*(**f**(*ξ*), **f**(**x**)) measures the intensity similarity. Both the closeness function and the similarity function are Gaussian functions of the Euclidean distance between their arguments. The filtered ROI can be seen in Figure 4(a).

### 2.5. LII Estimation

**Figure 5:**
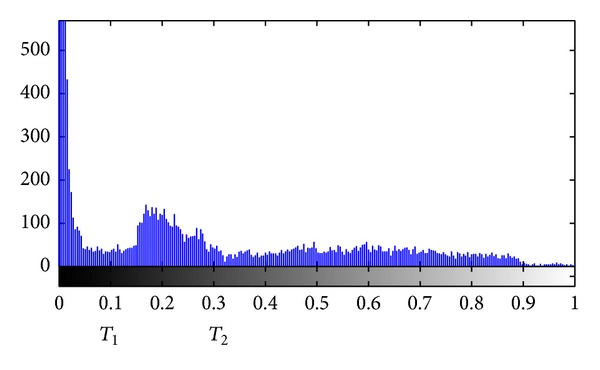
The intensity histogram of the filtered ROI.

After filtering, the intensity of the ROI is normalized into the scale of 0 to 1. When examining the histogram of the ROI intensity, we could find that the histogram consists of three peaks and two valleys, as shown in Figure 5. The two valleys are located at *T*_1_ and *T*_2_, respectively. A pixel with intensity between *T*_1_ and *T*_2_ may belong to the CCA intima, whereas pixels with intensity higher than *T*_2_ probably belong to the CCA adventitia. The value *T*_1_ can serve as the threshold to divide the pixels in the ROI into two clusters according to the intensity of each point. Suppose that the extracted ROI has a size of *M* lines and *N* columns; we need to find a point in each column in order to get a rough LII boundary. Inspired by Liu et al. [12], the rough LII was searched as follows.

1. Let *j* = 1; increase *i*_1_ from 1 to *M* until *I*(*i*_1_, *j*) > *T*_1_;(*i*_1_, *j*) is the starting point on the rough boundary.
2. Increase *j* to *j* + 1; search the first point *I*(*i*_2_, *j*) > *T*_1_ in the next column.
3. If *i*_2_ > *i*_1_, set (*i*_1_ + 1, *j*) as the next point on the rough boundary, if *i*_2_ < *i*_1_, set the next point at (*i*_1_ − 1, *j*), and if *i*_2_ = *i*_1_, select (*i*_1_, *j*) as the next point and then set vertical position of the new point as *i*_1_.
4. If *j* = *N*, output the rough boundary; otherwise, return to (2).

After the above operations, we can get an eight-neighborhood continuous line standing for the rough boundary of LII and a binary image segmented by the threshold *T*_1_. In some cases, intima layer may disappear at some places because of artifacts; thus, there would be a gap in the binary image, as shown in Figure 4(b). Morphological operations consisting of dilation followed by erosion were implemented to fill the gap. Finally we can get a flat line standing for the estimated LII boundary, as in Figure 4(c).

### 2.6. MAI Estimation

The boundary of MAI is estimated according to the LII, since the two lines are approximately paralleled to each other. Additionally, the MAI lies on the border of media and adventitia where a strong edge locates.

In order to estimate MAI boundary using the initial LII, we proposed a simple method. Firstly, the edge map of the ROI was computed; secondly, the initial LII boundary was displaced downward for several pixels taking into account the normal IMT range (0.4–1.4 mm) and the pixel density of the images; thus we got a series of parallel lines; and then the value of the edge strength on each line was summed up; at last, the line with the highest value was chosen as the estimated MAI boundary. The estimated LII and MAI can be seen in Figure 4(d).

### 2.7. Boundaries Adjustment with Dynamic Programming

Dynamic programming [26] is a technique for solving optimization problems when not all variables in the evaluation function are interrelated simultaneously. To formulate the boundary-snapping procedure as dynamic programming, we must define an evaluation function that embodies a notion of the “best boundary.” In our situation, both LII and MAI boundaries are nearly straight lines at the position of strong edges. Therefore, the “best boundary” can be denoted by a weighted sum of high cumulative edge strength and low cumulative curvature. For an *N*-segment curve boundary, the following formula can be established:

(4) $$\begin{array}{c} h\left(x_{1},x_{2},\ldots ,x_{N}\right)=\overset{N}{\underset{k=1}{\sum }}g\left(x_{k}\right)+\lambda \overset{N-1}{\underset{k=2}{\sum }}c\left(x_{k-1},x_{k},x_{k+1}\right), \end{array}$$

where *λ* is a negative constant, *x*_*k*_ is the point on the estimated boundary and can move upward or downward for a pixel, and the function *g*(*x*_*k*_) is the edge strength at position *x*_*k*_ and the function *c*(*x*_*k*−1_, *x*_*k*_, *x*_*k*+1_) is the curvature of the polyline defined by the 3 points: *x*_*k*−1_, *x*_*k*_,  and  *x*_*k*+1_. {*x*_1_, *x*_2_,…, *x*_*N*_}, the solution of the largest *h*(*x*_1_, *x*_2_,…, *x*_*N*_), can be found by applying dynamic programming, which consists of the points of the “best boundary.”

Canny [27] showed that Gaussian derivatives yield good compromise between localization and detection. Therefore, we convolved the ROI with a vertical directional first-order derivative of Gaussian kernel to get the edge map, which was previously used in Section 2.6. The Gaussian kernel we applied has a size of 10 × 10 with standard deviation of 1 in both horizontal and vertical directions. The vertical directional derivative of the Gaussian kernel has the advantage for detecting edges lying approximately in the horizontal direction.

**Figure 6:**
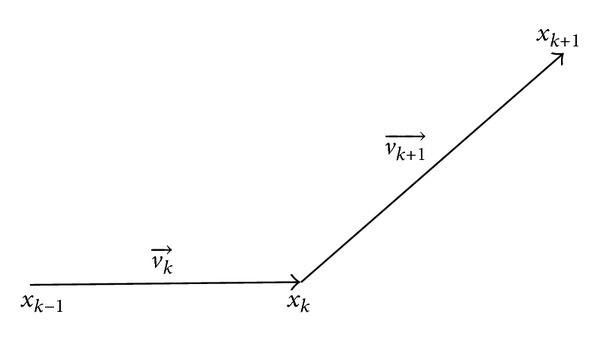
Estimation of the curvature.

Practically, curvature has become a basic tool to formulate a contour function. Williams and Shah's work [28] presented several curvature approximation methods for discrete curves and evaluated their efficiency of computation, accuracy of the estimation, and presence of anomalies. In a situation where a curve consists of three not evenly spaced points (Figure 6), their work proved that taking the difference between the normalized vectors could give a good estimation for the curvature; that is, the formula of the curvature is given by

(5) c(xk−1,xk,xk+1)=(vk→|vk→|−vk+1→|vk+1→|),2where,  vk→=xk−xk−1,  2≤k≤N−1.

After the dynamic programming procedure, we could get the accurate boundaries both for LII and MAI as shown in Figure 4(e).

### 2.8. IMT Measurement and Validation

The IMC lies almost horizontally in the ultrasound image, and the detected boundaries of LII and MAI have the same number of points. Therefore, the IMT can be measured by calculating the mean absolute distance between the two detected boundaries:

(6) $$\begin{array}{c} \text{IMT}=\frac{1}{N}\overset{N}{\underset{k=1}{\sum }}\left|\text{MAI}\left(k\right)-\text{LII}\left(k\right)\right|, \end{array}$$

where *N* is the number of points constituting the two boundaries and *k* is the index spanning the columns of the image. The maximum and minimum values of {|MAI(*k*) − LII(*k*)|, 1 < *k* < *N*} can be used to test whether the detected IMT is reasonable. If they are not within the range of 0.2 mm to 1.6 mm, the IMT measurement is failed.

When processing a large number of ultrasound images, the automatic segmented (AS) IMT values and ground truth (GT) IMT values should be compared. There are two important criteria for the comparisons: correlation and Bland-Altman plot.

The two sets of IMT values are correlated and the Pearson *R* coefficient is used to give an estimate of the measurement agreement:

(7) $$\begin{array}{c} R=\frac{\sum_{i=1}^{n}\left(\text{AS}\left(i\right)-\text{as}\right)\left(\text{GT}\left(i\right)-\text{gt}\right)}{\sqrt{\sum_{i=1}^{n}{\left(\text{AS}\left(i\right)-\text{as}\right)}^{2}}\sqrt{\sum_{i=1}^{n}{\left(\text{GT}\left(i\right)-\text{gt}\right)}^{2}}}, \end{array}$$

where *n* is the number of subjects for IMT measurement, AS(*i*) and GT(*i*) are the automatic segmented IMT values and ground truth IMT values for *i*th subject, and as and gt are the mean value of the two sets of IMT.

The mean and standard deviation (SD) between AS and GT IMT are plotted in the Bland-Altman plot. This method is very effective in pointing out possible biases in IMT measurement.

## 3. Results

In our experiment, the ground truth (GT) of the IMT was measured by expert vascular clinician, which was manually computed by delineating the LII and MAI boundaries on the ultrasound images for six times and then averaged. The automatic segmented (AS) IMT is measured by applying the proposed algorithm.  Table 1 shows the parameters' list in our experiment, where *d* is the positive constant integer in Section 2.3; *T*_1_ is the threshold in Section 2.5; *r* is the size of the structuring element applied to morphological operations in Section 2.5; *λ* is the weights of curvature in Section 2.7.

**Figure 7:**
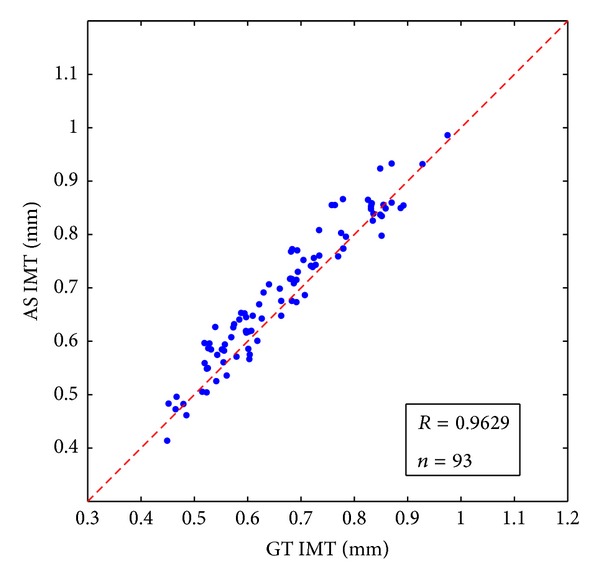
Automatic segmented IMT versus ground truth IMT.

**Figure 8:**
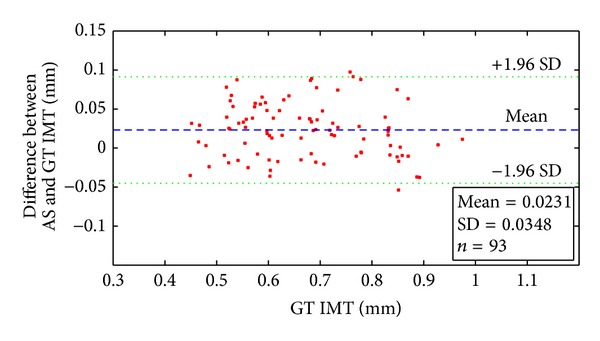
Bland-Altman plot between AS and GT IMT.

**Figure 9:**
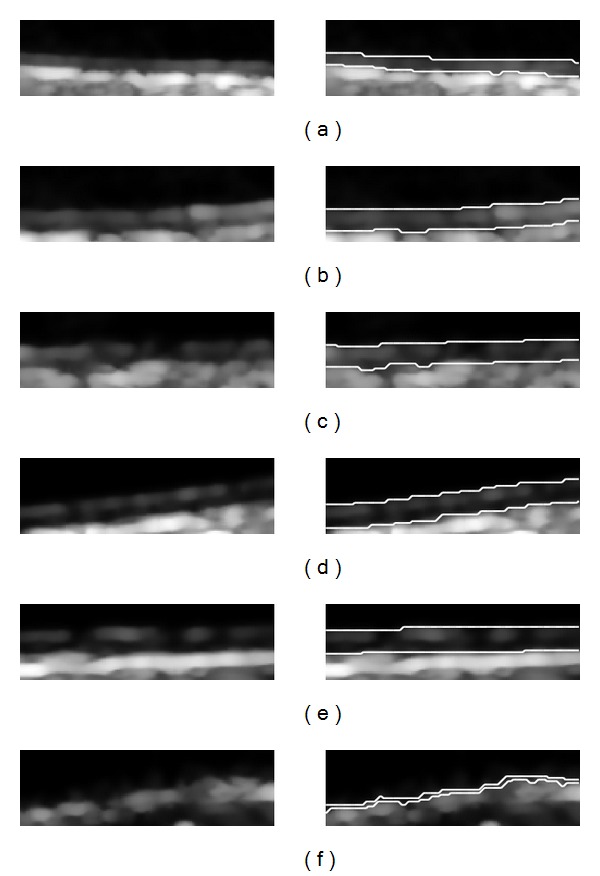
Examples of the extracted ROI and the final boundaries. The first five examples illustrate the correctly detected IMT and the last one shows the case of failed detection. In (c) and (e), the intima layer is incomplete. In (b) and (c), the adventitia layer is destroyed by noises or artifacts. In (f), the intima layer is completely disappeared and the IMT measurement is failed.

100 ultrasound images from 100 patients were tested by our algorithm. Seven images were failed for IMT measurement with our approach because of the absence of the intima layer. Among the 93 correctly detected images, the mean IMT ± standard deviation for GT and AS is 0.6707 ± 0.1275 mm and 0.6938 ± 0.1279 mm; the mean difference ± standard deviation between AS and GT is 0.0231 ± 0.0348 mm. The correlation coefficient is 0.9629 for AS and GT IMT. The average processing time for automated measurement is 0.3223 s with MATLAB under Windows XP on an Intel Core 2 Duo CPU E7500 @2.93 GHz.  Figure 7 shows the scatter diagram of the AS IMT with respect to GT IMT.  Figure 8 shows the Bland-Altman plots for AS IMT versus GT IMT.  Figure 9 shows another six pairs of the experiment results; the left sides of the pictures are the extracted ROI and the right halves are the final boundaries. The first five examples illustrate the correctly detected IMT and the last one shows the case of failed detection.

## 4. Discussions

In our experiment, the mean difference of AS and GT is 0.0231 mm among the 93 correctly tested images; that is, the IMT computed by our technique is more likely thicker than that manually measured by expert. This may be due to the ambiguous boundary understanding of MAI, while human did not delineate the concave contours according to gradient maxima. Figures 9(b) and 9(c) show the concave contours automatically detected by our approach.

The IMC template used in Section 2.3 should contain an IMC strip with a medium IMT value (about 0.7 mm). It can be cropped from one ultrasound image of the dataset and then smoothed by bilateral filtering. For a set of ultrasound images with the same pixel density and similar contrast, the template is quite robust for detecting IMC regions correctly. Even if some points (no more than four out of seven) are positioned at wrong places because of speckle noises or artifacts, the negative effects can be ignored.

**Figure 10:**
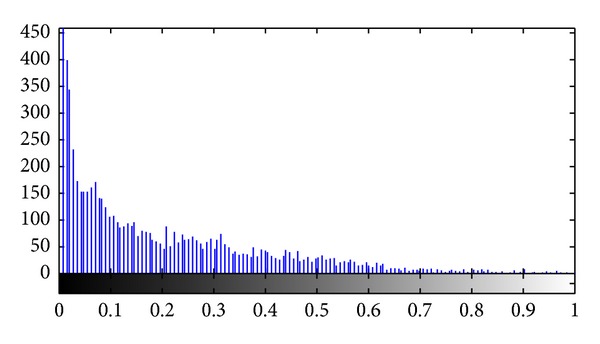
The intensity histogram of the failed ROI.

The threshold *T*_1_ in Section 2.5 can be adaptive according to the contrast of ultrasound images. The dataset used in our experiment comes from the same machine with the same parameters setting. Therefore the 93 images were correctly detected with *T*_1_ = 0.1. Among the seven failed images, the intima layer has completely disappeared. The ROI of IMC can be correctly detected, but the final LII and MAI boundaries are almost overlaid (Figure 9(f)). The intensity histogram of the failed ROI was shown in Figure 10. Compared to Figure 5, peaks and valleys are ambiguous in this histogram image.

Our approach is compared with some representative researches in Table 2. The results show low mean IMT bias and short processing time with other researches. However, there are three limitations related to the proposed approach. Firstly, the IMC template should be changed when the algorithm applied to ultrasound images from other machines because of different pixel density or image contrast. Secondly, the results of LII estimation are sensitive to plaques. Thirdly, the mean bias of LII and MAI were not investigated in our experiment for the lack of information on LII and MAI boundaries of manual tracing.

The proposed algorithm in this paper is especially suitable for medical uses, where some ultrasound equipment has been set to automatic IMT measurement for different patients. There are only two parameters that need to be set for a new machine, that is, the IMC-like template and the intensity threshold *T*_1_ for LII detection. After that the machine can be used for automatic IMT measurement from one patient to another. Since the processing time is less than 0.5 s per image in MATLAB, it has the potential to achieve real-time IMT measurement when using C++ under Visual Studio.

## Figures and Tables

**Table 1:** Parameters' list in our experiment.

Parameters	*d*	**T**_1_	*r*	***λ***
Values	20	0.1	15	−0.2

**Table 2:** Comparison with previous researches.

Previous researches	*n*	Mean IMT bias	Processing time	Automatic
Menchon-Lara et al. (2014) [21]	60	0.64 ± 0.19 mm	3.44 s	Yes
Molinari et al. (2012) [17]	365	0.078 ± 0.112 mm	<15 s	Yes
Petroudi et al. (2012) [19]	100	0.095 ± 0.0615 mm	21 s	Yes
Xu et al. (2012) [14]	50	0.0381 ± 0.0164 mm	0.43 s	No
Our approach	100	0.0231 ± 0.0348 mm	0.32 s	Yes
